# Ultra-Processed Foods and Nutritional Intake of Children and Adolescents from Cantagalo, São Tomé and Príncipe

**DOI:** 10.3390/children11091089

**Published:** 2024-09-05

**Authors:** Rita Morais, Mónica Rodrigues, Francisca Ferreira, Renata Barros, Patrícia Padrão, Madalena Ortigão, Maria Tavares, Pedro Moreira

**Affiliations:** 1 Faculty of Nutrition and Food Sciences, University of Porto, 4150-180 Porto, Portugal; up201810064@edu.fcna.up.pt (R.M.); up201711728@edu.fcna.up.pt (F.F.); renatabarros@fcna.up.pt (R.B.); patriciapadrao@fcna.up.pt (P.P.); 2 Epidemiology Research Unit, Laboratory for Integrative and Translational Research in Population Health, Institute of Public Health, University of Porto, 4050-600 Porto, Portugal; 3 Helpo–Non-Governmental Organization for Development, 2750-318 Cascais, Portugal; madalenaortigao@helpo.pt (M.O.); mariatavares@helpo.pt (M.T.)

**Keywords:** children, adolescents, ultra-processed foods, nutrients, São Tomé and Príncipe

## Abstract

Background: Globally, dietary patterns are shifting toward an increased consumption of ultra-processed foods, raising the risk of some metabolic and nutritional diseases from a young age. This trend is now also affecting low- and middle-income countries. Considering this, we aimed to assess the contribution of ultra-processed foods to total energy intake and their association with the nutritional intake of children and adolescents in Cantagalo, São Tomé and Príncipe. Methods: We conducted a cross-sectional study with a sample of 546 households. Data were collected on anthropometrics, sociodemographic characteristics, and lifestyle, including 24 h food recall questionnaires for children and adolescents. The reported foods were nutritionally assessed and categorized according to the NOVA classification to estimate the contribution of ultra-processed foods. Logistic regression models were used to estimate the magnitude of the associations between higher consumption of ultra-processed foods and nutritional intake, adjusting for confounders. Results: The contribution of ultra-processed foods to daily energy intake was 9.5% for girls and 8.5% for boys. Higher consumption of ultra-processed foods was significantly associated with a lower intake of fiber (OR = 0.932; 95%CI, 0.872–0.996), vitamin B12 (OR = 0.812; 95%CI, 0.668–0.985), and zinc (OR = 0.443; 95%CI, 0.308–0.639) and a higher intake of iron (OR = 1.479; 95%CI, 1.065–2.055) and sodium (OR = 1.001; 95%CI, 1.000–1.001), after adjusting for confounders. Conclusion: Higher consumption of ultra-processed foods was especially associated with a lower intake of fiber, vitamin B12, and zinc, and with a higher intake of iron.

## 1. Introduction

The Democratic Republic of São Tomé and Príncipe (STP) is a small archipelago formed by two islands, the São Tomé Island and the Autonomous Region of Príncipe, and by their adjacent islets. In 2022, there were about 219.0781 inhabitants, of which 41% were children and adolescents aged between 0 and 19 [1]. Regarding the socioeconomic context, STP is considered a low–middle-income country (LMIC), presenting a high level of poverty and income inequality [2]. The district of Cantagalo is part of the southeast region of the São Tomé island [1,3] and had around 19,000 inhabitants in 2017 [4], presenting a monetary poverty of 65.9% [3] and population density values below the country’s average [4].

Over the last few years, Sub-Saharan Africa has experienced two contrasting trends: a continuous increase in the prevalence of overweight and obesity alongside a persistently high prevalence of hunger and undernutrition [5,6]. This dual reality can be attributed to the ongoing global nutritional transition, which is contributing to the development of chronic non-communicable diseases (NCDs). This shift in dietary patterns not only exacerbates the prevalence of overweight and obesity but also places additional strain on African health systems. Consequently, the rising incidence of NCDs may further complicate public health efforts and strain healthcare resources across the continent [6].

The nutritional transition has paralleled the demographic transition, characterized by increased economic development and subsequent lifestyle and health status improvements. Concurrently, the epidemiological transition has seen a decline in infectious diseases. However, a rise in cardiovascular diseases, obesity, certain types of cancers, and type 2 diabetes has been emerging [7,8]. This shift in lifestyle and diet has also driven significant changes in food systems, largely due to advances in food processing. These changes have altered the structure, nutritional content, and flavor of foods while simultaneously increasing the availability, accessibility, and commercialization of ultra-processed foods (UPFs) [9]. Although there is no consensual definition for these foods, they are commonly described as industrial formulations composed of multiple food-derived ingredients and additives, including substances such as colorings, flavorings, and sweeteners, which are produced through a series of industrial processes [10,11,12]. Furthermore, UFPs are characterized by their high content of energy, saturated fat, sugar, and sodium, which in large quantities have been demonstrated to exert detrimental effects on health as well as contribute to poor dietary and nutritional quality [11,12,13]. 

This increase in UPF consumption seems to be one of the main causes of the obesity epidemic, which seems to be linked to the increased prevalence of hypertension, diabetes, and metabolic syndrome [9,14,15]. In fact, in Africa, studies have reported an increase in the prevalence of overweight and obesity associated with increased UFP consumption, leading to an increase in NCDs [16]. Among LMICs, São Tomé and Príncipe stands out as one of the nations with the highest prevalence of overweight and obesity among children, as well as one of the countries with the greatest overlap of undernutrition and overweight/obesity in this age group [17].

This dual burden underscores the crucial need to comprehend how UPFs are being incorporated into the dietary patterns of children in LMICs and how they may affect nutritional intake. Such efforts are essential for mitigating the prevalence of nutritional diseases and fostering improved health outcomes among vulnerable populations. 

To the best of our knowledge, there is no information regarding UPF contribution to children’s dietary intake in STP, as well as no studies on the association between UPF consumption and nutritional intake taking into consideration socioeconomic, demographic, and anthropometric characteristics. Taking this into consideration, the present study aims to evaluate UPFs’ contribution to total energy intake as well as their association with the nutritional intake of children and adolescents attending the second cycle of Basic Education (BE) in schools from the district of Cantagalo, in STP.

## 2. Materials and Methods

This cross-sectional study is part of the MeNutRic Plus project—Improvement of the Nutritional Status of Children in São Tomé and Príncipe, which is a program implemented by Helpo, a Portuguese Non-Governmental Organization for Development in the district of Cantagalo, in STP. 

This project was approved by the Health Ethics Committee for Scientific Research of the Democratic Republic of São Tomé and Príncipe and had a local partnership with the National School Food and Health Program supervised by the Ministry of Education of STP and a technical and scientific partnership with the Faculty of Nutrition and Food Sciences of the University of Porto (FCNAUP). The study was individually presented to all participants and their guardians. They were informed that participation was voluntary, that the collected data would remain confidential, and that participants would remain anonymous.

### 2.1. Participants Assessment

The sample size of students attending the 2nd cycle of BE in the district of Cantagalo was previously calculated by the National Institute of Statistics of STP (INE) following a convenience sampling method to ensure that the representative sample was equally distributed across all the schools in Cantagalo. In the initial phase, 573 children and adolescents from 8 public schools were assessed, and after analysis, 27 questionnaires were excluded due to incomplete information. Therefore, this study included a sample of 546 children, aged 9 to 16 years old, and their households.

Within the scope of this project, data were obtained by a multidisciplinary team of trained interviewers between November 2022 and February 2023 through the application of questionnaires applied indirectly to the guardians, which included questions on sociodemographic characteristics and questions regarding the children/adolescents to assess their anthropometric and lifestyle characteristics, including a 24 h food recall questionnaire. 

To guarantee the confidentiality of the data and the anonymity of each participant, a numerical code was assigned to each child and their guardian. Furthermore, only students in the 5th and 6th grades of basic schools covered by the project were eligible to participate in the study, and when the questionnaires were administered to the children, they had to be present at school and accompanied by their guardian. Although only children attending these school years participated, the range of the children was from 9 to 16 years old. While the number of children aged 13 to 16 was relatively small, the presence of children in this older age group may be attributed to factors such as grade repetition or the interruption and subsequent resumption of their education. These circumstances are not uncommon in the Cantagalo STP context, where socioeconomic challenges can impact educational trajectories.

### 2.2. Sociodemographic Characterization and Lifestyle

The sample was characterized using variables related to the main members of the household, such as sex, relationship to the guardian/degree of kinship, education level, and occupation. Still concerning the family, information regarding the number of household members, monthly family income, and the amount of income allocated to food was also used. Regarding the children, data were collected on their daily time of physical activity, sport, and/or active play, in minutes.

### 2.3. Anthropometry

Weight and height assessment was carried out by a team of trained nutritionists following the internationally recommended methodology [18]. Based on these data, as well as the sex and age, the z-scores for the child’s body mass index (BMI) were calculated. This calculation was carried out using the 1.0.4 version of the WHO Anthro Plus^®^ software [19], following the World Health Organization (WHO) reference curves [20].

### 2.4. Dietary and Nutritional Intake

To evaluate food intake, a 24 h food recall questionnaire was carried out in which all foods and drinks consumed by the children were recorded during the 24 h of the day before the questionnaire was administered, i.e., from the time they got up to the time they went to bed. This questionnaire was implemented to the children with their guardian present; however, only the children contributed to the reporting. The interviewers received prior theoretical and practical training, which covered the behavioral conduct and the methodologies to be adopted when applying the questionnaires. These questionnaires were subsequently analyzed, estimating the dose/portion ingested of all declared foods and drinks, using, whenever necessary, food composition tables (from West Africa and Portugal) [21,22], weight and portions manuals [23], local photo albums to quantify foods from STP [24], and direct reading of food labels; additionally, when clarification of the food records was necessary regarding any specific and/or local foods and drinks, trained researchers in STP supplemented the information to enhance the accuracy of coding and quantifying the reported food. 

Finally, using the 11.11.32 version of the Food Processor^®^ software from ESHA Research, USA, with updates from West African foods, the dietary information was converted into nutritional information for subsequent analysis of the intake of the children and adolescents. The nutritional analysis focused on macronutrients and 10 micronutrients—vitamins A, B12, C, and D and folate, calcium, iron, magnesium, sodium, and zinc—with recognized importance in growth and roles as sentinel nutrients in children [25].

As an additional measure of critical evaluation of intake data, the Goldberg method [26] was used to identify participants with inaccurate reports in the 24 h recalls. The cut-off points were calculated from the physical activity level and subsequently compared with the ratio between energy intake (EI) and basal metabolic rate (BMR), which is calculated using Schofield’s equations [27] for children, based on age, sex, height, and weight. 

The lower and upper limit cut-off points, corresponding to 0.87 and 2.75 for boys and 0.84 and 2.67 for girls, respectively, were then obtained with a 95% confidence level. Questionnaires whose ratio between EI and BMR was below or above the cut-off points were considered “under-reports” or “over-reports”, respectively, and those that were within the range were considered “plausible reports”. After the calculation, we observed 63 under-reports, 210 plausible, and 4 over-reports in boys, with 6 missing cases, while in girls, we verified the existence of 45 under-reports, 208 plausible, and 5 over-reports, with 5 missing cases. However, considering the economic and nutritional situation of families in STP, a new analysis was carried out and results obtained with the “plausible reports” were compared with those obtained when using all the questionnaires. As no statistically significant differences were found for the variables of interest, we decided to use all questionnaires.

### 2.5. Degree of Food Processing

All reported foods and drinks were classified taking into account the NOVA Food Classification System [28,29]. This system categorizes foods and beverages into four groups based on their degree of industrial processing. Group 1 consists of all unprocessed or minimally processed foods (e.g., natural, packaged, cut, chilled, or frozen vegetables, fruits, potatoes, and other roots and tubers; eggs; grains of wheat, oats, and other cereals), Group 2 includes processed culinary ingredients (e.g., butter; oils made from seeds, nuts, and fruits; white, brown, and other types of sugar and molasses obtained from cane or beets), Group 3 encompasses processed foods (e.g., canned or bottled legumes or vegetables preserved in salt or vinegar; fruits in sugar syrup; salted, dried, smoked, or cured meat or fish), and Group 4 comprises ultra-processed foods. The latter group is characterized by industrial formulations containing five or more ingredients, often many more. Examples of UPFs include soft drinks, packaged snacks (sweet or savory), ice cream, chocolates, confectionery products, industrially packaged bread, margarine and spreads, cookies, industrially produced sweet desserts (packaged or instant), breakfast cereals and cereal bars, energy drinks and energy bars, dairy beverages, sweetened and flavored yogurts, chocolate milk beverages, instant soups, sauces, infant formulas, transition milk, ready-to-eat or heat-prepared foods, processed meats, as well as foods and beverages primarily or entirely composed of sugar, oils, fats, or other substances not commonly used in homemade or artisanal culinary preparations.

To evaluate the contribution of UPFs to the children’s diets, the absolute and relative daily energy intake from this group of foods was subsequently estimated. Based on the median energy contribution of UPFs, the intake of these foods was categorized into two groups, low and high contribution, corresponding to energy intake below the median and at or above the median, respectively.

### 2.6. Statistical Analysis

The statistical processing of all obtained data was performed using version 29.0 of the IBM Statistical Package for Social Sciences (SPSS^®^) for Windows. A descriptive analysis of the variables was conducted using measures of central tendency (mean, median), measures of dispersion (standard deviation—SD), frequencies (relative and absolute), and percentiles. To assess the normality of cardinal variables, skewness and kurtosis coefficients were analyzed. Regarding statistical tests, the Mann–Whitney test, chi-square test for independence, *t*-tests, and binary logistic regression were used to evaluate the relationships between variables. Initially, a comparative analysis of nutritional intake was performed according to the UPF intake categories, adjusting for total energy intake [30]. Subsequently, a logistic regression was conducted, with the dependent variable being the energy contribution of UPFs according to Group 4 NOVA (below or above the median, <2.854 and ≥2.854, respectively). The independent variables included macronutrients and the sentinel nutrients [25], the intake of nutrients that varied significantly according to the degree of UPF consumption; child’s sex, age, BMI/age z-score, and daily time of physical activity, sport, and/or active play (in minutes); sex, education level, and occupation of the primary household members; household size; monthly family income; and the amount spent on food. For regression analysis, occupational data were aggregated into two categories: remunerated and non-remunerated, with non-remunerated defined as those not receiving payment. The logistic regression model also included energy intake and misreporting, calculated using the Goldberg method, as additional adjustment variables. A 95% confidence level was considered for all statistical analyses.

## 3. Results

The sample characteristics according to UPF intake are presented in Table 1; the sample consisted of 546 children (283 boys), with a mean age of 10.8 years (SD = 1.17), and mean walking time is 132.1 min (SD = 130.22). Regarding BMI, the children/adolescents have a mean BMI-for-age z-score of −0.66 (SD = 0.98), but 33.7% have some form of thinness, and 4.4% are overweight or obese. Children/adolescents with the highest consumption of UPFs are significantly younger and have a significantly higher BMI-for-age z-score, but there are no other significant differences according to categories in NOVA group 4 (Table 1). 

Regarding nutritional status, the prevalence of thinness (severe, moderate, and mild) is slightly lower among boys (33.5%) compared with girls (34.7%), while the combined prevalence of overweight and obesity is also higher among girls (5.7%) compared with boys (3.3%) (Table 2). 

Household characteristics did not differ significantly according to the children’s and adolescents’ UPF intake (Table 3). Among household members, 478 were female, and 398 were identified as the mother of the child/adolescent. The majority (60.6%) had completed only the first or second cycle of primary education and were primarily occupied as houseworkers (34.4%). Approximately 497 respondents identified as the primary caregiver. The average household size was 5.87 members (SD = 1.92). Most families had a monthly household income between 2000 and 3000 STN (31.6%), with the majority spending 100 to 200 STN (55.3%) on food.

The average reported daily intake was 1481 kcal ± 568.9 for girls and 1489 kcal ± 580.9 for boys (Table 4). The average contribution of UPFs to daily EI was 147 kcal ± 213.4 for girls and 134 kcal ± 204.3 for boys, corresponding to 9.5% and 8.5% of the total energy intake, respectively (Table 4). 

Regarding reported nutritional intake according to UPF consumption, the high UPF intake group reported significantly higher energy consumption. After adjusting for total reported energy intake, girls in the high UPF consumption group had significantly lower intakes of fiber; vitamins A, B12, and D; and magnesium, while their intake of sodium and zinc was significantly higher. In boys, the intake of vitamin C and magnesium was significantly lower, whereas their intake of sugars, zinc, and sodium was significantly higher in the high UPF consumption group (Table 5).

Binary logistic regression analysis (Table 6) indicates that the children/adolescents’ higher consumption of UPF is inversely associated with having a ”remunerated” family member. Regarding the association between UPF intake and nutritional intake, it was also found that children’s higher UPF consumption was significantly associated with lower intakes of fiber, vitamin B12, and zinc but higher intakes of iron and sodium, after adjusting for the previously mentioned variables (Table 6) This model accounts for approximately 35.5% of the variance observed in the dependent variable.

## 4. Discussion

To the best of our knowledge, this is the first work carried out in STP that estimates the contribution of UPFs to total energy intake and their association with socioeconomic, demographic, anthropometric, and nutritional variables. 

The results obtained in the present study show a mean contribution of UPFs to daily energy intake of 8.5% and 9.5% of the total energy intake for boys and girls, respectively. Additionally, higher consumption of UPFs is associated with a lower intake of fiber, vitamin B12, and zinc and a higher intake of iron and sodium regardless of energy intake; sex; age; daily time of physical activity, sport, and/or active play (in minutes); BMI; or the various socioeconomic and demographic characteristics of the households.

In children and adolescents from LMICs, UPF consumption seems to represent a lower percentage of total energy intake compared with high-income countries. Nonetheless, in LMICs these foods may still represent up to 38% of total energy intake [31,32,33,34]. In Brazil, UPF consumption by adolescents was 64% of the total energy intake [35], and in Australia, the United Kingdom, and the United States of America, these foods contribute from 47% up to 68% of the total energy intake of children and adolescents [31]. 

Considering the lower consumption of UPFs in LMICs, it is noteworthy that, despite their relatively modest percentage of total energy intake compared with other countries, especially in our study, UPFs already exhibit a negative association with crucial nutrients such as fiber, vitamin B12, and zinc. This association highlights the potential nutritional concerns associated with UPFs, as their intake may have a detrimental impact on the intake of important micronutrients for the prevention of metabolic diseases [36], even when at lower levels. Taking this into consideration, there is an evident need to tackle and decrease the rise in UPF consumption in LMICs, including Cantagalo. This necessity is underscored by the increasing availability and accessibility of UPFs in the market as well as their convenience and practicality, which tend to promote their purchase over more nutritionally beneficial options. However, as STP is more isolated from the rest of the African continent, it is possible that the availability and accessibility of UPFs ends up not being as great compared with other continents or even the rest of the African continent. Specifically, UPFs are often favored over foods rich in dietary fiber, such as fruits and vegetables [37,38,39], and these findings underscore the need for public health initiatives aimed at stopping or reducing UPF intake and promoting healthier food choices to improve childhood health outcomes, particularly regarding cardiometabolic risk factors [40]. Considering this, efforts should be directed toward implementing public health strategies to raise awareness and develop interventions designed to encourage healthier dietary choices among the younger population [37,38]. Additionally, some other studies have linked an increase in UPF consumption to a decrease in fiber intake, which is in line with the results obtained in our study [37,41]. This finding is concerning given that a diet high in fiber is associated with better health outcomes, such as improved blood glucose, insulin sensitivity, and lipid profile [37,38,42], and that this nutrient can be considered a strong indicator of diet quality [37,43,44].

Several studies indicate that the consumption of UPFs is positively associated with deficits in micronutrient intake, namely, vitamin B12 [13,45] and zinc [13,45,46], as their content in NOVA group 4 foods tends to be lower compared with other, less processed products [47]. In the case of vitamin B12, the results of this study are in agreement with the literature. A low consumption of foods rich in or fortified with vitamin B12 can lead to a deficiency of this vitamin, which can interfere with children’s growth and compromise neurological processes [48]. In relation to zinc, the same lower intake with higher UPF consumption can be seen, which may have negative consequences in children/adolescents’ health, considering that zinc inadequacy can compromise growth, cognitive health, and immune function [25] as well as increase the risk of developing metabolic and cardiovascular diseases [49,50]. 

A positive relationship was also observed between the consumption of NOVA group 4 foods and iron intake, conflicting with what many studies indicate, suggesting that UPFs are poor in this micronutrient [13,46]. However, a study carried out in Brazil also found an increase in iron intake with increased consumption of UPFs, which can be explained by the fortification of this nutrient in some of these foods [51], as may have happened in the present study. Furthermore, in our study, we may assume that the intake of UPFs may also include some charcuterie processed meat products (made with meat or blood from the animal), which may have contributed to an increase in iron intake.

In the present study, there was also an increase in sodium intake with the greater consumption of UPFs, but the higher odds were very low. Furthermore, there is a possibility that the amount of sodium consumed by children was underestimated due to the way it was calculated. Several studies show that previous 24 h food recall questionnaires may not be an accurate sodium measurement tool, not only due to the difficulty in knowing the amount of salt used during the meals [52], but also due to the scarcity of resources in LMICs to update sodium databases, which can lead to measurement errors [52,53]. Despite the possible underestimation of sodium intake, the results of the present study are in line with what several authors report when they associate UPFs with foods very rich in this micronutrient [31,54]. Even considering the low intensity of this association, these results are relevant, as elevated sodium intake during childhood has been linked to an increased risk of high blood pressure [55] and, consequently, an increased risk of developing cardiovascular diseases in adulthood [56,57]. 

The results of this study reveal that 33.7% of children have some form of undernutrition and 4.4% are overweight and obese. If we compare with data from other years in STP, we can see that, among girls, we went from 5.0% (2016) to 34.7% of thinness (mild, moderate, or severe), and from 21.7% (2016) to 5.7% overweight and obesity [58]. In boys, we went from 9.7% (2016) to 33.5% of thinness and from 10.1% (2016) to 3.3% of overweight and obesity [58]. The values found are discrepant with pre-pandemic data, bringing into consideration the lack of knowledge about the impact that COVID-19 may have had on the nutritional status of children and adolescents in these regions. A lot of countries, including African countries, suffered interruptions of food supply chains because of the pandemic, which may have led to reduced availability and accessibility of food and, consequently, to an increase in thinness among children. In previous data on STP collected by some of the authors of this study, working with Helpo (unpublished data), there is a prevalence of 4.5% of thinness, 5.5% risk of being overweight, and 1.0% of being overweight. Given these data, an evolution in nutritional status seems to be observed toward an increase in thinness and, simultaneously, overweight and obesity.

Finally, it was observed that children/adolescents from households with “remunerated” family members—defined as those engaged in remunerated work—demonstrated lower consumption of UPFs. This finding may suggest that, as indicated by some studies, families with lower socioeconomic status are more likely to purchase UPFs due to their affordability [59,60].

This study presents some limitations, such as the use of a single 24 h food recall questionnaire, which is a method focused on a short-term evaluation period, is incapable of capturing seasonal variations in food consumption, and is susceptible to various errors. These errors may include memory errors, inaccuracies in portion size estimation, and difficulties in identifying product brands [61,62]. Additionally, this method may provide an imprecise quantification of sodium intake, possibly leading to an underestimation [52,53]. Moreover, the cross-sectional design of the study further limits the ability to establish causal relationships between UPF consumption and nutritional intake [63]. Furthermore, we can also consider the reporting of daily physical activity a limitation, since given that it is self-reported by the child, it may be subject to some degree of bias. Finally, while the sample is representative of basic schools in Cantagalo, it may not fully reflect the broader context of STP.

Nevertheless, the study has notable strengths, such as the detailed collection of 24 h recall data using updated software that includes local products and recipes and the involvement of trained nutritionists to minimize bias. The 24 h food recall questionnaire method, being quick and retrospective, helps reduce behavioral changes that might occur due to the measurement process [64]. Additionally, intake estimates were adjusted using the Goldberg method to enhance data accuracy and reliability.

In the future, it would be important to investigate the consumption of UPFs in other districts of São Tomé and Príncipe, complementing it with multiple-day 24 h food recall questionnaires and other evaluation methods such as food frequency questionnaires, which can average out the day-to-day variability in diet that is not captured by the 24 h food recall by asking about the usual intake over a longer period of time. It would also be advantageous to investigate the relationship between this consumption and the increase in chronic non-communicable diseases as well as the influence of UPF consumption on children’s cognitive development and school performance. Furthermore, considering the fast-growing food industrialization, it would be valuable to understand the link between the consumption of UPFs and sustainability, with a focus on developing healthier and more sustainable alternatives to UPFs.

## 5. Conclusions

The present study showed that a higher contribution of UPFs to total energy intake is associated with a lower intake of fiber, vitamin B12, and zinc and a higher intake of iron and sodium. More studies are needed in STP to understand how the inclusion of UPFs in people’s dietary pattern is evolving and how this consumption can be influenced by the demographic and socioeconomic characteristics of the population. Subsequently, public health measures must be implemented to promote the adoption of healthy eating habits and to stagnate, or even decrease, the consumption of UPFs in children and adolescents.

## Figures and Tables

**Table 1:** Children/adolescents’ characteristics according to UPF intake.

Children/Adolescents’ Characteristics	Total	Low UPF Intake	High UPF Intake	*p*-Value
Sex, n (%)				0.123
Female	263 (48.2)	122 (44.7)	141 (51.6)	
Male	283 (51.8)	151 (55.3)	132 (48.4)	
Age, years, mean ± SD	10.82 ± 1.17	10.92 ± 1.22	10.72 ± 1.10	0.037
BMI (Kg/m^2^), mean ± SD	16.33 ± 1.93	16.27 ± 1.92	16.38 ± 1.95	0.238
Z-score BMI/age, mean ± SD	−0.66 ± 0.98	−0.72 ± 0.93	−0.61 ± 1.02	0.039
Categories of z-score BMI/age, n (%)				0.465
Severe thinness	5 (0.9)	3 (1.1)	2 (0.7)	
Moderate thinness	32 (5.9)	15 (5.6)	17 (6.3)	
Mild thinness	147 (27.3)	74 (27.4)	73 (27.1)	
Normal weight	331 (61.4)	173 (64.1)	158 (58.7)	
Overweight	19 (3.5)	2 (0.7)	17 (6.3)	
Obesity	5 (0.9)	3 (1.1)	2 (0.7)	
Daily time of physical activity, sport, and/or active play (in minutes), mean ± SD	132.13 ± 130.22	131.10 ± 124.11	133.14 ± 136.22	0.599

Low UPF intake—consumption below the median (<2.854).; high UPF intake—consumption above the median (≥2.854).; SD—standard deviation.; BMI—body mass index.

**Table 2:** Nutritional status by sex, according to the BMI-for-age z-score.

Nutritional Status	Female	Male	*p*
Nutritional Status Categories			0.072
Severe thinness, n (%)	0 (0.0)	5 (1.8)	
Moderate thinness, n (%)	20 (7.6)	12 (4.3)	
Mild thinness, n (%)	71 (27.1)	76 (27.4)	
Normal weight, n (%)	156 (59.5)	175 (63.2)	
Overweight, n (%)	11 (4.2)	8 (2.9)	
Obesity, n (%)	4 (1.5)	1 (0.4)	

**Table 3:** Household characteristics according to children’s and adolescents’ UPF intake.

Household Characteristics	Total	Low UPF Intake	High UPF Intake	*p*-Value
Sex, n (%)				0.364
Female	478 (87.5%)	243 (89.0%)	235 (86.1%)	
Male	68 (12.5%)	30 (11.0%)	38 (13.9%)	
Relationship of the guardian, n (%)				0.850
Mother	398 (72.9%)	205 (75.1%)	193 (70.7%)	
Father	50 (9.2%)	23 (8.4%)	27 (9.9%)	
Grandfather/grandmother	29 (5.2%)	12 (4.8%)	16 (5.9%)	
Uncle/aunt	35 (6.4%)	16 (5.9%)	19 (7.0%)	
Other	34 (6.2%)	16 (5.9%)	18 (6.6%)	
Education, n (%)				0.231
No education	13 (2.5%)	6 (2.3%)	7 (2.6%)	
1st or 2nd cycle of BE	321 (60.6%)	155 (58.5%)	166 (62.6%)	
3rd cycle BE or secondary	190 (35.8%)	103 (38.9%)	87 (32.8%)	
Higher education	6 (1.1%)	1 (0.4%)	5 (1.9%)	
Occupation, n (%)				0.344
Student	13 (2.4%)	5 (1.8%)	8 (3.0%)	
Houseworker	187 (34.4%)	91 (33.5%)	96 (35.4%)	
Farmer	19 (3.5%)	7 (2.6%)	12 (4.4%)	
State employee	66 (12.2%)	33 (12.1%)	33 (12.2%)	
Private/public company	24 (4.4%)	14 (5.1%)	10 (3.7%)	
Self-employed	156 (28.7%)	84 (30.9%)	72 (26.6%)	
Unpaid worker	27 (5.0%)	14 (5.1%)	13 (4.8%)	
Unemployed	45 (8.3%)	24 (8.8%)	21 (7.7%)	
Retired	6 (1.1%)	0 (0.0%)	6 (2.2%)	
Are you the primary caregiver? n (%)				0.654
No	49 (9.0%)	23 (8.4%)	26 (9.5%)	
Yes	497 (91.0%)	250 (91.6%)	247 (90.5%)	
Household members, mean ± SD	5.87 ± 1.92	5.94 ± 1.86	5.80 ± 1.98	0.671
Monthly household income, n (%)				0.631
<1000 STN	76 (17.8%)	41 (19.4%)	35 (16.2%)	
1000–2000 STN	113 (26.5%)	52 (24.6%)	61 (28.2%)	
2000–3000 STN	135 (31.6%)	70 (33.2%)	65 (30.1%)	
>3000 STN	103 (24.1%)	48 (22.7%)	55 (25.5%)	
Income spent on food, n (%)				0.507
<100 STN	159 (30.9%)	79 (31.0%)	80 (30.9%)	
100–200 STN	284 (55.3%)	135 (52.9%)	149 (57.5%)	
>200 STN	71 (13.8%)	41 (16.1%)	30 (11.6%)	

SD—Standard deviation.; BE—basic education; STN—São Toméan Dobras.

**Table 4:** Daily energy intake and energy contribution of UPF.

	Total	Female	Male
Energy (kcal)	1485.48 ± 574.67	1481.60 ± 568.94	1489.26 ± 580.93
UPF (kcal)	140.18 ± 208.64	146.75 ± 213.42	134.07 ± 204.28
UPF (%)	9.0 ± 12.6	9.5 ± 13.0	8.5 ± 12.3

**Table 5:** Nutritional intake by sex according to UPF consumption.

Nutritional Intake	Total	Low UPF Intake	High UPF Intake	Crude *p*-Value	Adjusted *p*-Value *
Girls					
Energy (kcal)	1481.40 ± 568.94	1370.67 ± 542.12	1577.22 ± 576.05	<0.001	
Total lipids (g)	51.62 ± 27.91	47.18 ± 28.01	55.27 ± 27.37	0.004	0.940
Saturated fatty acids (g)	9.90 ± 6.14	9.18 ± 5.15	10.51 ± 6.83	0.126	0.364
Total carbohydrates (g)	202.16 ± 81.20	187.73 ± 75.79	214.65 ± 83.88	0.003	0.916
Total sugars (g)	46.86 ± 37.19	39.99 ± 35.52	52.81 ± 37.69	0.002	0.081
Protein (g)	44.24 ± 17.60	40.55 ± 16.92	47.44 ± 17.62	<0.001	0.175
Dietary fiber (g)	14.11 ± 7.91	14.66 ± 8.26	13.63 ± 7.58	0.340	0.046
Vitamin A (µg)	147.15 ± 122.84	157.39 ± 121.28	138.29 ± 123.93	0.092	0.021
Vitamin B12 (µg)	3.95 ± 3.48	4.23 ± 3.65	3.70 ± 3.32	0.138	0.010
Vitamin C (mg)	61.61 ± 49.87	63.39 ± 52.33	60.07 ± 47.78	0.349	0.068
Vitamin D (µg)	7.99 ± 7.20	8.61 ± 7.54	7.44 ± 6.87	0.110	0.004
Folate (µg)	101.02 ± 82.49	97.20 ± 87.37	104.33 ± 78.19	0.169	0.979
Calcium (mg)	215.15 ± 148.05	198.09 ± 122.64	229.91 ± 165.97	0.275	0.991
Iron (mg)	4.57 ± 2.16	4.31 ± 2.01	4.79 ± 2.27	0.047	0.898
Magnesium (mg)	151.89 ± 65.28	155.15 ± 68.95	149.06 ± 62.04	0.713	0.002
Sodium (mg)	1489.46 ± 1076.12	1107.20 ± 580.66	1820.22 ± 1279.94	<0.001	<0.001
Zinc (mg)	3.50 ± 1.77	3.45 ± 1.71	3.54 ± 1.82	0.640	0.015
Boys					
Energy (kcal)	1489.26 ± 580.93	1379.38 ± 479.49	1614.95 ± 658.32	0.004	
Total lipids (g)	48.97 ± 27.25	43.37 ± 22.91	55.36 ± 30.32	<0.001	0.091
Saturated fatty acids (g)	9.07 ± 5.38	8.22 ± 5.14	10.04 ± 5.49	<0.001	0.208
Total carbohydrates (g)	210.01 ± 87.21	198.93 ± 77.13	222.69 ± 96.22	0.046	0.074
Total sugars (g)	47.50 ± 42.99	39.52 ± 41.56	56.61 ± 42.93	<0.001	0.032
Protein (g)	43.86 ± 17.14	40.46 ± 14.13	47.74 ± 19.37	0.002	0.168
Dietary fiber (g)	13.29 ± 8.97	13.05 ± 8.32	13.56 ± 9.68	0.980	0.231
Vitamin A (µg)	147.50 ± 131.93	148.12 ± 137.20	146.78 ± 126.14	0.864	0.442
Vitamin B12 (µg)	4.07 ± 3.53	4.07 ± 3.37	4.07 ± 3.72	0.602	0.090
Vitamin C (mg)	63.57 ± 61.11	63.94 ± 56.43	63.14 ± 66.28	0.252	0.040
Vitamin D (µg)	8.76 ± 7.65	8.53 ± 7.04	9.03 ± 8.31	0.978	0.246
Folate (µg)	98.90 ± 67.18	100.83 ± 76.79	96.69 ± 54.35	0.967	0.098
Calcium (mg)	211.32 ± 179.97	201.77 ± 76.79	222.24 ± 157.55	0.047	0.494
Iron (mg)	4.43 ± 2.23	4.22 ± 1.58	4.67 ± 2.78	0.363	0.067
Magnesium (mg)	154.62 ± 73.76	155.31 ± 62.21	153.83 ± 85.33	0.219	<0.001
Sodium (mg)	1367.02 ± 977.04	1062.09 ± 603.90	1715.86 ± 1186.41	<0.001	<0.001
Zinc (mg)	3.54 ± 1.77	3.50 ± 1.62	3.59 ± 1.94	0.990	0.001

* Adjusted for total energy intake.

**Table 6:** Association between UPF consumption (NOVA group 4) and nutritional intake *.

	High UPF Intake	*p*-Value
Sex of child/adolescent (Ref.: male)	0.994 (0.589; 1.677)	0.981
Age of child/adolescent	0.964 (0.756; 1.230)	0.769
Z-score BMI/age	1.147 (0.888; 1.480)	0.294
Daily time of physical activity, sport, and/or active play (min)	1.000 (0.998; 1.002)	0.871
Sex of household members (Ref.: male)	0.535 (0.247; 1.159)	0.113
Education (Ref.: not studied, 1st or 2nd cycle EB)	0.651 (0.372; 1.139)	0.132
Occupation (Ref.: non-remunerated)	0.579 (0.337; 0.993)	0.047
Household size	0.925 (0.796; 1.075)	0.307
Monthly household income (Ref.: <1000 STN)		0.692
1000–2000 STN	1.319 (0.556; 3.127)	0.529
2000–3000 STN	0.991 (0.438; 2.238)	0.982
>3000 STN	1.452 (0.560; 3.767)	0.443
Amount dispensed for food (Ref.: <100 STN)		0.709
100–200 STN	0.848 (0.437; 1.643)	0.624
>200 STN	0.675 (0.266; 1.712)	0.407
Total lipids (g)	0.913 (0.809; 1.031)	0.168
Saturated fatty acids (g)	1.053 (0.974; 1.138)	0.353
Total carbohydrates (g)	0.976 (0.928; 1.027)	0.382
Total sugars (g)	1.003 (0.996; 1.011)	0.141
Protein (g)	1.043 (0.982; 1.108)	0.196
Fiber (g)	0.932 (0.872; 0.996)	0.036
Vitamin A (µg)	1.000 (0.997; 1.003)	0.970
Vitamin B12 (µg)	0.812 (0.668; 0.985)	0.035
Vitamin D (µg)	0.971 (0.894; 1.055)	0.488
Folate (µg)	0.996 (0.991; 1.001)	0.122
Iron (mg)	1.479 (1.065; 2.055)	0.020
Sodium (mg)	1.001 (1.000; 1.001)	0.001
Zinc (mg)	0.443 (0.308; 0.639)	<0.001

* The logistic regression was additionally adjusted for misreporting and energy intake.

## Data Availability

Data are unavailable due to privacy or ethical restrictions.
